# The uses and adverse effects of beryllium on health

**DOI:** 10.4103/0019-5278.55122

**Published:** 2009-08

**Authors:** Ross G. Cooper, Adrian P. Harrison

**Affiliations:** 1 Physiology Division, Birmingham City University, Egbaston Campus, 030 Bevan House, Westbourne Road, Edgbaston, Birmingham B15 3TN, UK; 2 Section for Biochemistry & Physiology, Department of Animal & Veterinary Basic Sciences, Faculty of Life Sciences, Copenhagen University, 1870 Frederiksberg C, Denmark

**Keywords:** Beryllium, environment, exposure, fumes, health, inhalation, occupation

## Abstract

**Context:**

This review describes the health effects of beryllium exposure in the workplace and the environment.

**Aim:**

To collate information on the consequences of occupational and environmental exposure to beryllium on physiological function and well being.

**Materials and Methods:**

The criteria used in the current review for selecting articles were adopted from proposed criteria in The International Classification of Functioning, Disability, and Health. Articles were classified based on acute and chronic exposure and toxicity of beryllium.

**Results:**

The proportions of utilized and nonutilized articles were tabulated. Years 2001–10 gave the greatest match (45.9%) for methodological parameters, followed by 27.71% for 1991–2000. Years 1971–80 and 1981–90 were not significantly different in the information published and available whereas years 1951–1960 showed a lack of suitable articles. Some articles were published in sources unobtainable through requests at the British Library, and some had no impact factor and were excluded.

**Conclusion:**

Beryllium has some useful but undoubtedly harmful effects on health and well-being. Measures need to be taken to prevent hazardous exposure to this element, making its biological monitoring in the workplace essential.

## INTRODUCTION

Beryllium (Be) mostly occurs naturally as beryllium aluminium silicate (beryl), 3BeO.Al_2_O_3_.6SiO_2_. As a naturally occurring element, beryllium is present in rocks, coal and oil, soil and volcanic dust.[1] The element forms a light, hard, noncorrosive metal.[2] Although beryllium was discovered in 1798, it only became commercially important in the 1930s. Of the excess of 40 beryllium-bearing minerals known, only beryl and bertrandite are commercially important. Beryl which contains ca. 11% beryllium oxide is mostly mined in Brazil and Russia.[3] Impurities in beryl include alkali metals, alkaline earth metals, iron, manganese, and phosphorus. Gems include emeralds (beryl-containing chromium), aquamarine (beryl-containing iron), and other semiprecious stones.[4] Bertrandite (4BeO. 2SiO_2_.H_2_O) is the main form mined in the USA (Colorado, New Mexico, and Utah) despite containing < 1% beryllium; it can be processed into beryllium hydroxide.[4–5] Although no adverse clinical effects have been observed in gemstone cutters working with beryls, workplace hygiene is essential along with biological monitoring of beryllium in the urine.[6]

Beryllium is a Group II metallic element with a silver-grey-whitish color, an atomic weight of 9.01, melting point of 1,287°C, boiling point of 2,970°C, and density of 1.85 at 20°C.[3] Its hexagonal crystalline structure makes it the lightest of all solids and chemicals, and it has a very high melting point, specific heat, heat of fusion, and strength-to-weight ratio.[3] It is lighter than aluminium and over 40% more rigid and ca. 33% more elastic than steel.[3] It is insoluble in water and soluble in acids and alkaline solutions. It has extremely good electrical and thermal conductivity and is not magnetic. At ordinary temperatures, beryllium resists oxidation in the air and a thin layer of beryllium oxide on its surface makes it highly resistant to corrosion.

Pure beryllium metal is used to make components in aircraft disc brakes, X-ray transmission windows, space vehicle optics and instruments, aircraft and satellite structures, missile parts, nuclear reactor neutron reflectors, nuclear weapons, fuel containers, precision instruments, rocket propellants, navigational systems, heat shields, mirrors, high-speed computers, and audio components.[3]

Beryllium chloride exists as white-to-colorless, deliquescent crystals, soluble in water, alcohol, benzene, ether, chloroform, and carbon disulphide, and insoluble in ammonia and acetone.[3] Beryllium chloride is used mainly to make beryllium metal by electrolysis, and is used as an acid catalyst in organic reactions.[3] Beryllium fluoride exists as a colorless amorphous mass, soluble in water and slightly soluble in alcohol, and is used in glass-making and nuclear reactors.[3] Beryllium hydroxide exists in three forms (i) metastable, tetragonal, crystalline solid, ii) stable, orthorhombic, crystalline solid, and iii) a slimy, gel with a slightly basic pH).[3] It is soluble in acids and alkalis but insoluble in water. Beryllium fluoride and hydroxide are used to produce beryllium metal and alloys.[3] Beryllium oxide occurs as a white powder or gel that is insoluble in hot water and soluble in acids, alkalis, and ammonium carbonate.[3] Beryllium nitrate is used as a chemical reagent and for stiffening mantles in gas and acetylene lamps. Beryllium oxide is a very commonly used substance in high-technology ceramics, electronic heat sinks, electrical insulators, microwave oven components, gyroscopes, military vehicle armor, rocket nozzles, crucibles, nuclear reactor fuels, thermocouple tubing, lasers, in high-density electrical circuits, automotive ignition systems, and as an additive to glass, ceramics, and plastics[4], in the preparation of beryllium compounds, as a catalyst for organic reactions, and in high-temperature reactor systems.[3] Beryllium metaphosphate is a white porous powder or granular material that is insoluble in water.[3] It is used as a raw material in special ceramic compositions and as a catalyst carrier. Beryllium orthophosphate is soluble in water and acetic acid.[3] Beryllium sulphate occurs as colorless crystals that are insoluble in cold water and alcohol but decompose in hot water.[3] Beryllium sulphate is used mainly for the production of beryllium oxide powder for ceramics. Beryllium sulphate tetrahydrate occurs as colorless crystals that are soluble in water, virtually insoluble in ethanol, and slightly soluble in concentrated sulphuric acid.[3] It is used as a chemical intermediate in the processing of beryl and bertrandite ores. Beryl ore occurs as colorless, blue-green, yellow or white, transparent, hexagonal crystals that are insoluble in acid.[3] Beryllium-copper alloy usually contains 4–4.25% beryllium by weight, with a melting point of 870–980°C and produces toxic fumes of beryllium oxide upon heating. Beryllium-copper alloy is used in electrical connectors and relays, wheels and pinions, nonsparking tools, and switches in cars.[7] Beryllium-aluminium alloy contains 20–60% beryllium[3] and has been used in the manufacture of light aircraft and casting alloys.[3]

Bertrandite and beryl are mined commercially for the recovery of beryllium ore, with very pure gem-quality beryl being known as aquamarine (blue or blue-green) or emerald (green).[1] As a component of alloys, beryllium contributes hardness, strength, and high electrical and thermal conductivity, and enhances resistance to corrosion, wear, and fatigue.[4]. An addition of 2% beryllium to copper increases copper's strength six-fold.[4] As an alloy, it is used in electrical and electronic parts, as construction material for machinery and moulds for plastics, automobiles, computers, sports equipment, e.g., golf clubs and bicycle frames, dental bridges,[1] nuclear reactors, aerospace, ceramics,[2] springs, precision instruments, nonsparking tools, submarine cable housings and pivots, wheels, pinions, telecommunications devices etc.[4] Dental technicians are exposed to beryllium and various other dusts and chemicals and, as a consequence, are at high risk of developing Chronic Beryllium Disease (CBD) and other lung conditions.[8–10] One study described how a dental technician developed pulmonary granulomatosis; also, a diagnosis of pneumoconiosis is related to exposure to beryllium and aluminium.[11]

Beryllium was determined to be a lung hazard in Europe in the 1930s soon after its production in modern industry.[12] It was only in the 1940s that the USA recognized lung diseases associated with beryllium exposure[13] in workers engaged in manufacturing fluorescent lamps.[14] Exposure of workers to beryllium may occur via metal machining, recycling beryllium from scrap alloys, and using beryllium products, being ca. 21,000 in 2002.[1] Pure beryllium metal is used in nuclear weapons and reactors, aircraft and space vehicle structures, instruments, X-ray machines, and mirrors.[1] Primary production of beryllium metal which was used in nuclear weapons components, resulted in cases of severe dermatitis, reversible pneumonitis, and chronic granulomatosus lung disease.[15–16] CBD provides a human model of pulmonary granulomatous disease produced by occupational exposure, occurring more frequently in those with a genetic predisposition. It can be differentiated from sarcoidosis by specific immunological testing.[17–18]

In nuclear energy sites using beryllium, workers may experience significant exposure during maintenance, repair, renovation, or demolition activities.[19] Acute pneumonitis cases may progress chronically to irreversible granulomatous lung disease, especially when working with copper-beryllium alloys.[13] Beryllium oxide is also made from beryllium ores and is used to construct ceramics for electrical and high-technology applications.[1] Delayed effects of CBD following exposure to beryllium ceramic products have an incidence of 2.9–15.8%.[20]

Absorption of beryllium is principally via the lungs. Beryllium depositing in the lungs, moves exceedingly slowly into pulmonary blood circulation. During the natural clearing of the throat and cilliary mucus clearance, beryllium may enter the mouth and be swallowed but fortunately, < 1% is absorbed into the gastrointestinal capillaries. It becomes protein-bound and deposits over a long period in the liver, spleen, and skeleton despite a portion being excreted by the kidneys;[5] its urinary excretion is variable. Acutely it causes pneumonitis, cough, chest pain, dyspnea, and pneumonia.[2] Conjunctivitis, rhinitis, and pharyngitis are common and it is a skin irritant. Chronically, it results in sarcoid-like granulomata mainly in the lungs and is occasionally subcutaneous. Lung lesions often result in progressive interstitial fibrosis with hilar lymphadenopathy with resultant cor pulmonale. Beryllium is a likely candidate as a lung carcinogen.[2,21] Beryllium compounds cause genetic transformations in cultured mammalian cells resulting from binding of ionic beryllium to nucleic acids, resulting in infidelity of DNA replication.[22] Lung cancer is likely during exposure with an excess relative risk of 1.2–1.6.[3] Acute beryllium pneumonitis is associated with higher lung cancer rates as a marker for high exposure with relative risk of up to 2.3.[23] Lung cancer incidence is especially increased among workers (standardized mortality ratio (SMR) of 3.33) with a history of acute beryllium disease associated with very high beryllium exposure.[24] Lung cancer is increased among those with acute disease (SMR = 2.32) than in those with chronic disease (SMR = 1.57).[23]

The effects of inhaled cigarette smoke (active and passive) cannot be excluded. Beryllium metal and beryllium-aluminium alloy, beryl ore, beryllium chloride, beryllium chloride, beryllium hydroxide, beryllium sulphate tetrahydrate, and beryllium oxide potentiate the growth of lung tumors in rats exposed by either a single intratracheal instillation or a one-hour inhalation exposure.[3] Other animal studies include the growth of anaplastic carcinoma following exposure to beryllium oxide and beryllium sulphate in monkeys after intrabronchial implantation or inhalation.[3] Osteosarcoma has been induced in rabbits exposed to beryllium metal, beryllium carbonate, beryllium oxide, beryllium phosphate, beryllium silicate, or zinc beryllium silicate by intravenous injection and/or bone implantation.[3]

Immunologically mediated beryllium hypersensitivity involves the accumulation of leukocytes around beryllium deposits to form granulomas and may occur 10–15 years following exposure to soluble or insoluble forms (> 5 × 10^−5^ mg/m^3^ air).[1] Both CBD and sensitization were found to occurr in former workers whose mean daily working lifetime average exposures were lower than the current allowable Occupational Safety and Health Administration workplace air level of 2 *μ*g/m^3^ and the Department of Energy guideline of 0.2 *μ*g/m^3^.[25] Sensitization and CBD were associated with areas in which beryllium air levels exceeded 0.2 *μ*/m^3^ and not with areas where this level was rarely exceeded. Employees at a particular copper-beryllium alloy facility had similar prevalence of sensitization and CBD as workers at facilities with higher beryllium air levels.[26]

Chest radiography may reveal widespread nodules that are 1–5 *μ*m in diameter and often coalesce. The Kveim test distinguishes berylliosis from sarcoidosis. Treatment involves the nonspecific management of pulmonary fibrosis. Chelation therapy using ethylenediaminetetraacetic acid (EDTA) may be used in acute poisoning and corticosteroids may be helpful in chronic fibrotic lung disease.[2] Hypersensitivity pneumonitis, a granulomatous interstitial disease of the lungs, results due to immune reactions following chronic inhalation of antigens in a presensitized person.[27] Diagnosis, based upon associated clinical and paraclinical signs include inspiratory crackles and bronchoalveloar lavage; high-resolution computed tomography scans are needed to confirm the diagnosis.[27] Beryllium was detected in the lungs of all Be-exposed subjects, with the highest levels of persistent beryllium inside CBD lung granulomas.[28] Beryllium antigen persistence may help explain the chronicity of this granulomatous disorder.[28]

Beryllium is sampled onto cellulose acetate filters (pore size 0.8 *μ*m) at an air flow rate of ca. 1 L/min and analyzed using atomic absorption spectrophotometry (30 min at 0.025 mg/m^3^). Workplace exposure limits are 8 h Time Weighted Average (TWA), 2 *μ*g/m,^3^ a value legally adopted by the Occupational Safety and Health Administration, USA. This standard was deemed to be protective against acute beryllium pneumonitis and for chronic beryllium disease. Sporadic cases were said to be due to incidents such as accidental spills.[13]

The aim of this article was to critically review the most important aspects of beryllium and their effects on human health, particularly from the context of workplace and environmental (including landfill) exposure.

## MATERIALS AND METHODS

The criteria used in the current review for selecting articles to be included were both theoretically and practically motivated and adopted from proposed criteria in The International Classification of Functioning, Disability and Health–ICF.[29] These criteria were as follows:

- Articles were chosen only with internationally recognized impact factors greater than 0.10.
- Articles were chosen based on the impact of lifestyle, stress, and/or environmental factor(s) predisposing beryllium exposure.
- Criteria for selection of literature included yes-no responses to: the appropriateness of methodology, adequacy of subject numbers, specificity of sex and/or age of subjects, and statistically significant response rates to survey questionnaires.
- The time frame used was principally 1931–2010, subdivided into nine-year categories, although articles of extreme importance from earlier decades were used wherever appropriate.
- A multifactorial overview concerning zinc exposure was elucidated. It was presumed that collective articles detailing known factors of usage were not necessarily correlated with functionality and health.
- Compilation of materials for the review started with published literature or easily accessible academic research.
- The articles were accessible from online sources including PubMEd and Medline.

## RESULTS

The annual subdivision of articles by year are presented in Table 1. The timeframe of 2001–10 has the greatest match (45.9%) based on methodological parameters, followed by 27.71% for 1991–2000. Years 1971–80 and 1981–90 were not significantly different and years 1951–1960 showed a lack of suitable articles. Some articles were published in sources unobtainable through requests at the British Library and some had no impact factor and were excluded.

**Table 1:** Selected results for articles on influence of beryllium and its environmental, ecological, and health effects

Time period	Total # journal articles sourced	Journals included	Journals excluded	Total # health brochures and books included
1931–1940	1	1	0	0
1941–1950	3	3	0	0
1951–1960	0	0	0	0
1961–1970	20	1	19	0
1971–1980	34	3	31	0
1981–1990	40	3	37	1
1991–2000	105	25	80	3
2001–2010	173	68	105	9
Total	376	104	272	13
			(6 excluded)	(13 utilized)

### Public health effects of beryllium and the workplace

Estimates of the number of US workers who were occupationally exposed to beryllium were published in the 1970s and 1980s, and ranged from 21,200 to 800,000.[30] As many as 134,000 current workers in the government and in private industry are potentially exposed to beryllium in the United States. We recommend that the results of this study be used to target atrisk audiences for hazard communications intended to prevent beryllium sensitization and chronic beryllium disease.[30]

Industries using beryllium in their products include aerospace, automotive, biomedical, defence, energy and electrical, fire prevention, instruments, equipment and objects, manufacturing, sporting goods and jewellery, scrap recovery and recycling, and telecommunications.[13] Examples of beryllium exposure in trivial, unrecognized, or brief exposures have been reviewed by Kreiss *et al*.[13] Beryllium is extremely toxic especially via the lung route, often damaging the mucosal lining and causing pneumonia and berylliosis, a dangerous and persistent lung disorder that can potentate damage to other organs including the cardiovascular system.[31] Allergic reactions may result in people who are hypersensitive to it, resulting in extreme cases of CBD. Classic symptoms include weakness, tiredness and breathing difficulties, associated anorexia, and bluish discolouration of the hands and feet, sometimes resulting in death[31] occasionally via cardiac enlargement and heart disease.[1] Beryllium-induced carcinogenesis is also a strong possibility.[31] The EPA estimated that the chance of developing cancer as a consequence of lifelong beryllium exposure of 4 × 10^−6^ mg/m^3^ air is 1:1,000.[1] Corticosteroids may be a useful means for controlling the symptoms of shortness of breath and delay the onset of heart failure.[5] Review of medical records of residents surrounding a beryllium facility showed that probable causes of CBD either displayed an abnormal blood test for beryllium and radiographic evidence consistent with disease, or met epidemiological criteria for CBD based on beryllium case registry criteria.[32]

The most serious sites in the USA, the National Priorities List (NPL), where hazardous waste leaves one exposed to beryllium, have been identified and documented by the Environmental Protection Agency (EPA).[1] The Federal Government targets such sites for massive clean-up operations, with 535 out of 1,613 sites testing positive for beryllium in 2002.[1] The American Conference of Governmental Industrial Hygienists (ACGIH) researches the risk factors, exposure metrics, and natural history of CBD.[33]

Beryllium exposure in non-smokers via passive smoking is of concern. One study showed that increased lung cancer among workers with higher lagged beryllium exposure and lack of evidence for confounding by cigarette smoking, strongly suggest that beryllium is a human lung carcinogen.[34–35] More studies are needed to investigate the fetal exposure to beryllium in utero and in breast milk. Very young children may be exposed to beryllium via soil eating and unwashed hands.[1,36] It is not known whether children differ from adults in their susceptibility to beryllium and if exposure to beryllium will result in birth defects or other developmental effects in people.[37] It is likely that beryllium can be transferred from the mother to an infant through breast milk and/or that it can cross the placenta, but further studies are required to confirm this association.[37] Children are shorter than adults and may breathe dust, soil, and vapors close to the ground to a greater extent.[36] A child's lower body weight and higher intake rate results in a greater dose of hazardous substance per unit of body weight.[36] Children are dependent on adults for access to housing, for access to medical care, and for risk identification.[36] Thus, adults need as much information as possible to make informed decisions regarding their children's health.[36]

Workers in beryllium-processing facilities are encouraged to shower and change their clothes before they return to their families. Indeed, incidental exposure pathways may involve hand-to-mouth exposure, dermal contact, and resuspension following deposition of beryllium onto clothing.[38] Additionally, the element may be found in domestic consumer products including electronic devices such as nonsparking tools, televisions, calculators, and personal computers, although only deliberate manipulation of such will result in exposure.[1]

Process-related risks were associated with physiochemical properties of the exposed materials, determining the degree of beryllium bioavailability and immunologically mediated responses.[13] Preventative measures targeted these high-risk places, regardless of whether exposure was related to the regulatory standard.[13] However, reductions in airborne beryllium exposure did not prevent sensitization and measures were explored to limit the number of workers in high-hazard jobs, particle migration control, and the addition of dermal protection to respiratory masks and face shields.[13]

Job loss and stress and immunocompromisation are linked leading one to become more susceptible to lung infections, and thus a greater risk of beryllium damage.[16] Wound contamination may result in problems and skin protection is an important consideration. Synthetic beads of up to 1 *μ*m in diameter could move from the human skin surface into the dermis where immunologically active cells are found.[39] Even with the implementation of control measures to reduce skin contact with beryllium as part of a comprehensive workplace protection program, measurable levels of beryllium continue to reach the skin of workers in production and production support areas.

Based on our current understanding of the multiple exposure pathways that may lead to beryllium sensitization and CBD,[40] support of prudent control practices such as the use of protective gloves to minimize skin exposure to beryllium salts and fine particles, is necessary.[41]

Insoluble beryllium oxide particles placed onto the skin surface of laboratory mice result in an immunological reaction when the mice were subsequently challenged by dermal application of beryllium salt.[39] Glove use and other measures including cleaning contaminated clothing are important aspects of beryllium exposure prevention. Included in the like are the removal of contaminated clothing, avoiding gaps between clothing and gloves and other areas of exposed skin. Beryllium contamination of the hands under occlusive gloves was associated with beryllium-contaminated shoes and respirators prior to wearing the gloves.[42] Further studies on the chronic effects of beryllium exposure by concentrations accumulated in a particular surface area every 48 hours would be useful to gauge the association between concentration exposure and pathology.

In a relatively recent investigation of steel workers in Kaohsiung, an important industrial area in Taiwan, Horng and colleagues[43] showed that urinary levels of beryllium were 1.58 ± 0.46 *μ*g/L whilst that of quality control workers were likewise high (1.58 0.46 *μ*g/L) when compared with values for control individuals (0.83 ± 0.46 *μ*g/L). The urinary beryllium level that is considered to be normal is < 2 *μ*g/L, and Horng and colleagues[43] only found evidence of two production workers with beryllium values > 2 *μ*g/L, one of whom showed signs of dermatitis, and the other, weakness. In dental technicians exposed to 0.04–1.70 *μ*g/m^3^ beryllium, urinary levels reached average levels of 0.34 *μ*g/L vs. controls (0.26 *μ*g/L).[44] More work is needed to show that the evaluation of urinary beryllium levels is a suitable tool for determining occupational exposure to the metal.[45]

## BERYLLIUM AND THE DIET

A major cause of chemical pollution still remains the discharge of wastewater that originates from either domestic use or industry. The risk of toxic material transfer, including metals, to the general population remains where drinking water is derived from sources that could have been affected by wastewater discharge (e.g., desalination plants or deep wells). In a study of the metal content of drinking water in the city of Riyadh, water samples were collected from 101 houses and of these, two were found to have a beryllium content of 1.05 *μ*g/L[46] which is far higher than the 0.19 *μ*g/L contamination found in a USA study.[1] In a study of 88 wines from four regions (Pfalz-Bad Dürkheim, Pfalz-Landau, Rheinhessen-Ingelheim, and Rheinhessen-Dienheim in Germany), the authors[47] concluded that beryllium content was on average 1.27 *μ*g/L (min. 0.08 and max. 3.20 *μ*g/L) which represents a “contamination” level that is some six times higher than that of the aforementioned USA study.[1] Thus, metals may form an elemental fingerprint of the origin of a wine, being influenced as they are by the soil composition of the vineyard.[47] At present, no health-based guideline values exist for beryllium in drinking water. Other dietary sources of beryllium may include marine organisms,[48] as it has been shown that plankton concentrate beryllium. As plankton are the food source of other marine organisms, including the ascidians (sea quirts–phylum *Chordata*, class *Ascidiacea*), there is a real risk of further concentrating this metal. Indeed, Ishikawa and colleagues[48] reported abnormally high concentrations of beryllium in the livers of ascidians. Various Ascidiacea are used as food, among them the Sea pineapple (*Halocynthia roretzi*) which is cultivated in Japan and Korea and when eaten raw, is reputed to taste like “rubber dipped in ammonia,” apparently an acquired taste.

A less exotic risk of contamination may be posed by living close to a commercial incinerator of hazardous waste, as reported in a study from Tarragona County in Catalonia, Spain[49] where a new hazardous waste incinerator was built in 1996. As the risk of exposure to most metals by the general population is higher by food consumption than through inhalation, food samples obtained from local markets were analyzed for beryllium content. Bocio and colleagues[49] found that whilst some metals (As, Cd, Hg, and Pb) were consumed by the Tarragona County population at elevated levels in the local foods, beryllium levels were found to be below the limits of detection (0.05 *μ*g/g of food). Moreover, a 1998–2003 follow-up autopsy study of individuals (n = 22) found no detectable beryllium in the brain, bone, kidney, liver, or lungs of this Catalonian population[50] as a result of living close to such a commercial incinerator of hazardous waste.

## BERYLLIUM AND THE ENVIRONMENT

An NPI ranking is assigned to a substance that has health and environmental effects following exposure, and beryllium ranked 76 out of 400 with a total hazard score of 4.0.[51] On a health hazard rating of 0–3 (1: harmful to health, 2: medium hazard, 3: very high hazard), beryllium and its compounds rate 2.3.[51] In the USA, the average concentration of beryllium is 0.03 ng/m^3^ of air, increasing in cities to ca. 0.2 ng/m^3^ of air due to combustion of coal and fuel oil.[1] Beryllium enters waterways from the natural wearing away of rocks and soil.[1] Air and water effluent contaminated with beryllium and combustion of coal and oil result in its accumulation in the environment.[1,31] Beryllium compounds exist principally as fine dust particles in the air which eventually settle over land and water.[1] Extremely small beryllium particles may remain suspended in the air for up to ten days.[1] A study by the EPA found the most beryllium detected at any one time was 2 millionths g/m^3^.[1] Precipitation and snow remove beryllium from the air. It usually settles in sediment in waterways and may consequently be ingested by mud-sucking fish like *Clarias gariepinus* living near major effluent sites or in isolated waters like farm dams or quarries. Thus, beryllium accumulates in the subcutaneous fatty tissue of these fish and can possibly find its way into humans who consume the fish, particularly in developing countries.[52–53] Tissue samples would need to be collected and assayed for heavy metal concentrations before such an assumption becomes fact. Some beryllium is suspended in muddy water.

Drinking water usually has extremely low levels of beryllium, being found in 2002 to contaminate 5% of 1,577 samples in the USA with ca. 190 ng/L.[1] The EPA has determined in various parts of the USA, that the drinking water concentration of beryllium is < 2 trillionths g/L water.[1] Insoluble beryllium compounds remain in the ocean for a few hundred years before settling to the bottom of the seabed.[1]

Beryllium is found in various concentrations in the soil, although typical levels are ca. three thousandths g/kg.[1] Beryllium compounds may stay in the soil for thousands of years. Disposal of coal ash, incinerator ash, and industrial wastes may increase the amount of beryllium in the soil.[1] People who live in close proximity to hazardous landfill sites containing beryllium, may develop beryllium toxicity. Although beryllium levels have increased in the soil through dumping of industrial waste, the metal is unlikely to leach into ground water. Beryllium often reacts with dissolved chemicals, resulting in a less toxic, insoluble form which has little effect on aquatic organisms.[31] However, water-insoluble beryllium compounds may revert to soluble forms with consequent greater threats to human health.[1] In 2002, the concentration of beryllium in raw carrots and field corn was < 25 *μ*g/kg.[1] However, whilst the presence of calcium in soils has been shown to enhance the uptake of some inorganic elements (e.g., K and Rb) by root crops, there is no evidence of selective uptake of beryllium in the presence of calcium.[54] Beryllium content in fruit and fruit juices has been measured at levels ranging from < 0.1 *μ*g/L in a pineapple, to 74.9 *μ*g/L in a papaya.[3] Some fruits and vegetables accumulate beryllium, especially pears and kidney beans. This source, however, is unlikely to result in the metal accumulating in the bodies of animals that consume them,[31] as most of it is excreted rapidly in the urine and feces.[1] Deliberate ingestion of soluble beryllium salts may cause ulceration in dogs and skin scrapings may result in granulomatous rashes or ulcers.[1] Contact with broken skin may result in the formation of itchy ulcers and lumps or nodules on the exposed parts of the body two weeks after exposure.[5] Accidental injection of beryllium metal into the skin may result in the appearance of a granuloma.[5]

## TESTING FOR BERYLLIUM SENSITISATION

The Agency for Toxic Substances and Disease Registry[55] summarizes the salient points utilized in a blood testing program, the beryllium lymphocyte proliferation test (BeLPT), to determine sensitivity parameters to beryllium exposure. There is a vast plethora of published articles on BeLPT and we selected the ones with highest impact. BeLPT can detect antibodies produced by previous beryllium exposures. The test does have its limitations as the results are not always consistent or stable.[13,56] Laboratories performing the BeLPT or other similar biological assays of immunological response, could benefit from a statistical approach such as SPC to improve quality management.[57] The BeLPT is efficacious in medical surveillance of beryllium-exposed individuals. The positive predictive value of the BeLPT is comparable to other widely accepted medical tests. Confirmation of an abnormal result is recommended to assure appropriate referral for CBD medical evaluation.[58] BeLPT can result in false negatives and therefore, requires bronchoscopy, an invasive procedure for certain diagnosis of CBD.[59] The blood BeLPT should be used serially in beryllium disease surveillance to capture new or mixed cases of sensitization and disease within 50 days of initial exposure.[60] Beryllium sensitization does not occur when the level of beryllium at work is < 0.01*μ*g/m^3^.[61] Worker populations that have had the BeLPT test can be used to determine genetic associations and dysregulated inflammation during progression of disease.[62]

The USA allows testing in workers exposed to high levels of the metal, particularly those i) working in machine shops and via household contacts, ii) living within one-quarter of a mile of a beryllium-processing plant; and/or iii) having a diagnosis of sarcoidosis.[2] Lung damage usually follows inhalation of > 1 mg soluble beryllium/m^3^ of air.[1] General practitioners may use the BeLPT test to determine if patients have developed CBD. Classically, BeS, an immunologically mediated allergic reaction, may develop at random, shortly following exposure or years later. Some do not develop any adverse symptoms. CBD may develop in sensitized individuals exposed to small quantities of the metal. Classic symptoms of CBD include cough, shortness of breath, fatigue, fever, night sweats, appetite loss, and weight loss.[2] Sarcoidosis is very similar to CBD although beryllium sensitivity is absent. The time lag between clinical diagnosis of sarcoidosis and the final diagnosis of CBD ranged from zero to 18 years (median: three years) and the mean (range) age at the time of diagnosis of CBD was 43.9 (25–80) years. Beryllium-contaminated workplaces causing disease encompassed a wide spectrum of industries and technical trades in which beryllium exposure is generally not perceived as a health hazard. In conclusion, chronic beryllium disease still belongs to the spectrum of differential diagnoses of granulomatous disorders.[63]

Doctors usually advise patients testing positive to completely avoid further exposure to beryllium emissions. Within test limitations, the risk of developing CBD is low if the BeLPT test determines that one is not sensitized.[2] Beryllium sensitization and CBD mostly occur as a result of exposures in excess of 0.4 *μ*g/m^3^ and maintaining exposures below 0.2 *μ*g/m^3^ 95% of the time may prevent these conditions in the workplace.[64]

Chronic beryllium disease may be diagnosed via outpatient bronchoscopy with bronchoalveolar lavage and transbronchial biopsy.[13] Subclinical sensitizations devoid of lymphocyte alveolitis and alveolar granulomatosus are at high risk for development of CBD.[13] The concentrations of alveolar-deposited particles < 10 *μ*m in size and the number of alveolar-deposited particles smaller than 3.5 *μ*m may be useful for predicting CBD and sensitization.[65]

Questions have arisen over the avoidance of additional lung exposure of beryllium and its effectiveness in preventing lung disease.[13] Indeed, workers persistently exposed to beryllium in factories by working with beryllium oxide, alloy and/or metal are constantly at risk of lung disease. Usually, due to side-effects of the treatment regimes, treatment of beryllium disease with corticosteroids or other immunosuppressive therapy is delayed until abnormalities are detected.[66] The response to long-term corticosteroids in CBD, quite like that in sarcoidosis, is variable.[67] Significant lung function improvement may be seen following cessation of beryllium exposure.[67]

One study in Elmore, Ohio, USA, provided an opportunity for individuals who wanted to be tested for immune sensitivity to beryllium, albeit limited to i) concerned individuals living within 1.25 miles of the Brush-Wellman facility in Elmore, Ohio (the facility), ii) their household contacts, iii) employees of local machine shops that contract to machine beryllium alloys and their household contacts, and iv) individuals diagnosed with sarcoidosis.[68] Progressors to CBD were more likely to have worked as machinists.[69][70] The highest concentration of beryllium is on the floor of the driver's side of such workers' vehicles, suggesting that beryllium is carried on the soles of workers' shoes.[71]

Increased cumulative and individual lifetime-weighted exposure to total and respirable beryllium must be closely monitored.[72] In a study at a former nuclear weapons' facility, machinists (11.4% of total) and health physics technicians (11.9% of total) were found to have the greatest sensitization.[73] We found no difference in average age, sex, race or ethnicity, smoking status, or beryllium exposure time between those who progressed to chronic beryllium disease and those who remained sensitized without disease.[70] We conclude that beryllium sensitization is an adverse health effect in beryllium-exposed workers and merits medical follow-up.[70] Although current releases to the ambient air are not considered hazardous, little is known about the fate of beryllium that was: released to the air and deposited since 1953, incidentally taken home by the facility's beryllium workers, or incidentally taken home by workers at machine shops contracting with the facility to machine beryllium alloys.[68]

## GENETIC ASPECTS OF BERYLLIUM EXPOSURE

Genetic susceptibility studies in the workplace are useful as they provide a mechanistic insight into the etiology of the disease and the gene response, identify susceptible subpopulations that are exposed, and provide valuable input in setting occupational exposure limits.[74] Differential susceptibility in chronic beryllium disease has a genetic component and the associated immunologically mediated response involves activation of human leukocyte antigens (HLAs).[13] Beryllium-induced proliferation of T lymphocytes from the lungs of CBD patients are associated with HLA-DP alleles possessing a glutamic acid in the 69^th^ position of the B1 chain of this molecule.[75–76] The DP alleles that present beryllium to disease-specific T cell lines match those implicated in disease susceptibility.[77] The angiotensin-converting enzyme (ACE) genotype is important in the immune response to beryllium and in progression to beryllium disease,[78] and may reflect the extent of pulmonary granulomatous inflammation in CBD.[79] Self-presentation by T cells in response to beryllium can occur *ex vivo*, in the absence of professional antigen presenting cells, with a specific dependence on T cell-expressed major histocompatibility complex class II molecules and exogenous interleukin 2 for survival.[80] Analysis of the alleles reveals antigen-binding grooves and that subgroups with the greatest electronegativity have the most pronounced associations with chronic beryllium disease status.[81] There is a strong association between CBD and possession of alleles of HLA-DP containing glutamic acid; T cell clones were raised that secreted interferon-γ,[82] indicating a precise immunologically mediated mechanism. A mouse hybrid macrophage cell line, H36.12j, provides a useful tool for evaluating the mechanisms by which beryllium stimulates macrophage cytokine production and by which T cell-derived interferon-γ amplifies TNF-α production in granulomatous diseases.[83] When alleles were grouped by the relative negative charge on the molecules for which they code, the data suggest that those alleles associated with the most negatively charged proteins carry the greatest risk of beryllium sensitization and disease.[81] The frequency of beryllium-specific T cells in the blood of beryllium-exposed subjects may be a useful biomarker that helps to discriminate between beryllium sensitization and progression to CBD.[84]

It is possible that the combination of computational chemistry, genetics, and epidemiology will be useful for identifying a genetic test for high-risk HLA-DPB1^Glu69^ alleles.[13] Indeed, the association of CBD with HLA-DPB1 alleles that contain glutamine at position 69 occurs in 97% of people with CBD and 30–40% of beryllium-exposed controls.[85] The usefulness of HLA-DP in conferring susceptibility and the fact that residue 69 of HLA-DPB1 may be a predictor of CBD has been emphasized.[86] However, difficulties surrounding the interpretation of the HLA-DPB1-Glu^69^ marker, the lack of assurance regarding the protection of worker confidentiality, and lack of implementation of mandatory worker screening, combined to make testing beryllium workers impossible.[87] The expression of HLA-DPGlu^69^ determines higher T cell proliferation rates and more consistent *in vitro* responses to beryllium in the BeLPT test, both in the homozygous and the heterozygous states, possibly leading to an underestimation of the numbers of HLA-DPGlu^69^-negative, sensitized subjects within exposed populations.[88] Susceptibility for CBD has been associated with HLA-DP alleles possessing a glutamic acid at the 69th position (Glu^69^) of the β-chain. The mechanism for this association lies in the ability of these HLA-DP molecules to present beryllium to pathogenic CD_4_^+^ T cells. These findings in CBD have important implications for studies in autoimmune diseases, including those in which the inciting antigen is unknown and the target organ is inaccessible.[89]

There is no association between specific TNF-α alleles and either chronic beryllium disease or sensitization to beryllium.[90] Beryllium exposure induces transcription-dependent TNF-α production as a result of upregulation of specific transcription factors.[91] TNF-α production-associated polymorphisms are carried at a higher frequency by beryllium hypersensitivity-affected persons compared to beryllium-exposed controls, suggesting an important role of TNF-α in the former.[92] Sensitization and CBD are multigenetic processes and the specific genes involved interact with exposure and determine the risk of the disease.[93] Beryllium-induced human lung adherent macrophage apoptosis may contribute to the re-exposure to beryllium, resulting in chronic granulomatous inflammation.[94] Beryllium stimulates macrophage reactive oxygen species formation which plays an important role in Be-induced macrophage apoptosis.[95] Beryllium-stimulated macrophage cell line apoptosis is caspase-dependent and not solely dependent on beryllium-stimulated TNF-α levels, and the release of beryllium antigen from apoptotic macrophages results in the re-introduction of beryllium material back into the alveolar sacs.[96] This would make it available for uptake by fresh macrophages and result in the amplification of beryllium-stimulated cytokine production, resulting in progressive inflammation and granuloma formation.[96]

The immunological behavior of T lymphocytes following beryllium exposure has been elucidated in terms of toxicokinetic and toxicodynamic effects, e.g., 70% of the total beryllium (10–100 ng/g serum was detected in prealbumin[97] and clinical studies of acute beryllium disease).[98] The programmed death pathway (PD-1) is active in beryllium-induced disease and plays a key role in controlling beryllium-induced T-cell proliferation.[99] Beryllium mediates a thiol imbalance leading to oxidative stress that may modulate the proliferation and clonal expansion of CD_4_^+^ T cells.[100] CBD is associated with the ability of certain HLA-DP molecules with associated peptides to bind and present beryllium to pathogenic CD_4_^+^ T cells.[101] Beryllium-ferritin exposure may potentiate persistent antigen production which is associated with the chronicity of CBD and its development years after environmental beryllium exposure has ceased.[102] Balance between interleukin-10 (IL-10) and IL-6 release and the associated p45 phosphorylation are essential components of beryllium-mediated immune responses.[103] There is a great increase in antigen-specific effector memory CD_4_^+^ T cells in the lungs of CBD patients.[104] The presence of circulating beryllium-specific CD_4_^+^ T cells directly correlates with the severity of lymphocytic alveolitis. The findings in CBD have important implications for studies in autoimmune diseases, in particular, those with an unknown inciting antigen and an inaccessible target organ.[105] It an help to construct a model in which beryllium stimulation of peripheral blood mononuclear and dendritic cells can modulate the expression and release of different chemokines, leading to the migration of lymphocytes to the lung and the formation of a localized environment for development of Be disease in susceptible individuals.[106] The functional capability of antigen-specific CD_4_^+^ T cells is determined by the relative proportions of memory T cell subsets, which may reflect internal organ involvement.[107]

Ethical considerations in genetic testing cannot be ignored. However, issues of confidentiality arise particularly if employers have access to employee genetic profiles.[13] Additionally, insurance charges for covering company-foreseen genetic susceptibility to chronic beryllium disease would be massive.[13] However, new employees would benefit from genetic testing as they would be afforded the choice of weighing their risk tolerance in working in the beryllium industry.[13] Beryllium acts as a potent pharmacological inducer of premature senescence in human fibroblasts.[108]

## DISCUSSION

According to our research parameters, there has been clearly a drive in the last decade to investigate occupational exposure to contaminants like beryllium. The gap of relevant articles from 1951 to 1960 suggests that health concerns of workers took a back seat during the time of profiteering and industrialization in the USA. Like with any metal, the consequences of long-term exposure to beryllium should be taken seriously. Clearly a comprehensive exposure preventative program will reduce beryllium sensitization in new workers during the first years of employment.[42] Problems of increased risk result from the differing physicochemical characteristics of different beryllium compounds and aerosol exposure.[13] Several aerosol sampling devices are available, nominally at least, for each of the conventions. During machining, more than 50% of beryllium particles in the breathing zone are < 10 *μ*m in aerodynamic diameter, contributing to beryllium deposition into the deepest recesses of the lung.[109] Some considerations have already been discussed that are important to the sampling of airborne particles containing beryllium with regard to the sampling conventions, the test protocols, and sampler performance.[110] One study emphasized the need to investigate aerosol exposure to beryllium from particles on fabric fibres resuspended from garments.[111]

The authors emphasized the need for increased management commitment to health and safety, empowerment of employees to maintain a clean workspace, and training videos on methods of exposure risk.[13] In a copper-beryllium alloy processing facility, workers were exposed to beryllium in the greatest concentrations exceeding 0.2 *μ*g/m^3^.[26] The overall prevalence of beryllium sensitization and CBD for workers in these three copper-beryllium alloy distribution centers is lower than for workers in primary beryllium production facilities.[112] CBD resulting from exposure to low-beryllium content copper demonstrated restrictive lung and low diffusion capacities, and a biopsy specimen showed epithelioid cell granulomas and alveolitis.[113]

Government public health regulations should be enforced via inspections of beryllium-handling plants, hazardous landfill sites, and food and drinking water testing, in conjunction with public health facilities. Limits of exposure, conforming with international agreements, e.g. the EPA, should be enforced in workroom air with releases of < 0.01 *μ*g/m^3^ of air averaged over a 30-day period, and a maximum allowable limit of 4 × 10^−3^ mg/L in drinking water.[1] Engineering controls are the best way of reducing exposure to beryllium via enclosure of operations and/or local exhaust ventilation to reduce dust; beryllium preparations should be transported as liquids, not powders.[5] Personal protective equipment (respirators, masks, protective eyewear, clothing, and gloves) is also important.[5] The mass of the respirable particles, their chemical form, and particle surface chemistry may be associated with the prevalence of CBD.[33] Assessing beryllium exposure by all routes of exposure including inhalation, dermal uptake, and ingestion will add useful information to current knowledge.[33] Additionally the form of beryllium may affect the likelihood of developing CBD as the exposure to beryl and bertrandite ore dusts or beryllium salts, in the absence of exposure to beryllium oxide particulates, appears to pose a lower risk for developing CBD.[38]
